# Supplementary material for: Habitat Imaging of Tumors Enables High Confidence Sub-Regional Assessment of Response to Therapy
Source: Cancers (Basel). 2022 Apr 26;14(9):2159. doi: 10.3390/cancers14092159 (PMC9101368; doi:10.3390/cancers14092159)
Supplement: Supplementary file 1 [file cancers-14-02159-s001.zip › cancers-1648619-supplementary.pdf]

Article

Habitat Imaging of Tumors Enables High Confidence Sub-Regional Assessment of Response to Therapy

Paul David Tar [1], Neil A. Thacker [2], Muhammad Babur [3], Grazyna Lipowska-Bhalla [1], Susan Cheung [1], Ross A. Little [1], Kaye J. Williams [3] and James P. B. O'Connor [1,4,5,*]

1 Division of Cancer Sciences, University of Manchester, Manchester M13 9PT, UK; paul.tar@manchester.ac.uk (P.D.T.); grazyna.lipowska-bhalla@manchester.ac.uk (G.L.-B.); susan.cheung@manchester.ac.uk (S.C.); ross.little@manchester.ac.uk (R.A.L.)

2 Division of Informatics, Imaging and Data Sciences, University of Manchester, Manchester M13 9PT, UK; neil.thacker@manchester.ac.uk

3 Manchester Pharmacy School, Division of Pharmacy and Optometry, University of Manchester, Manchester M13 9PT, UK; muhammed.babur@manchester.ac.uk (M.B.); kaye.williams@manchester.ac.uk (K.J.W.)

4 Department of Radiology, The Christie Hospital NHS Trust, Manchester M20 4BX, UK

5 Department of Radiology, The Christie Hospital NHS Trust, Manchester M20 4BX, UK

* Correspondence: james.oconnor@icr.ac.uk

Abstract: Imaging biomarkers are used in therapy development to identify and quantify therapeutic response. In oncology, use of MRI, PET and other imaging methods can be complicated by spatially complex and heterogeneous tumor micro-environments, non-Gaussian data and small sample sizes. Linear Poisson Modelling (LPM) enables analysis of complex data that is quantitative and can operate in small data domains. We performed experiments in 5 mouse models to evaluate the ability of LPM to identify responding tumor habitats across a range of radiation and targeted drug therapies. We tested if LPM could identify differential biological response rates. We calculated the theoretical sample size constraints for applying LPM to new data. We then performed a co-clinical trial using small data to test if LPM could detect multiple therapeutics with both improved power and reduced animal numbers compared to conventional t-test approaches. Our data showed that LPM greatly increased the amount of information extracted from diffusion-weighted imaging, compared to cohort t-tests. LPM distinguished biological response rates between Calu6 tumors treated with 3 different therapies and between Calu6 tumors and 4 other xenograft models treated with radiotherapy. A simulated co-clinical trial using real data detected high precision per-tumor treatment effects in as few as 3 mice per cohort, with p-values as low as 1 in 10^{-4}. These findings provide a route to simultaneously improve the information derived from preclinical imaging while reducing and refining the use of animals in cancer research.

Keywords: Cancer, Imaging, Modelling, Statistics, Machine Learning, ADC

1. Statistical significances of biological responses

Table S1. Statistical significances of biological responses represented as Z-Scores and equivalent p-values for Control Calu6 tumor and model.

tumor	V1-V2 Z	V1-V2 P	V1-V3 Z	V1-V3 P	V1-V2-V3 Z	V1-V2-V3 P
Control						
1	0.2	0.834	1.4	0.154	1.6	0.111
2	0.0	1.000	0.0	0.972	0.0	0.991
3	0.3	0.780	0.2	0.821	0.1	0.951
4	0.3	0.752	0.3	0.782	0.3	0.740
5	0.2	0.871	0.4	0.725	0.5	0.651
6	0.0	0.967	1.1	0.291	0.5	0.598
7	1.5	0.140	0.8	0.432	0.9	0.364
8	0.0	1.000	1.3	0.208	1.2	0.249
9	0.0	1.000	0.0	0.966	0.3	0.764
10	0.0	1.000	0.1	0.942	0.6	0.579
11	0.1	0.953	0.2	0.875	0.0	1.000
12	1.5	0.138	0.0	0.986	0.2	0.853
13	0.5	0.582	0.0	0.998	0.0	1.000
14	0.0	1.000	1.9	0.055	0.1	0.960
15	0.3	0.766	0.0	1.000	0.2	0.813

Table S2. Statistical significances of biological responses represented as Z-Scores and equivalent p-values for RT Calu6 tumor and model.

tumor	V1-V2 Z	V1-V2 P	V1-V3 Z	V1-V3 P	V1-V2-V3 Z	V1-V2-V3 P
RT						
1	3.2	0.001	5.2	$2.19E^{-7}$	8.6	$>1.00E^{-16}$
2	0.7	0.498	5.5	$4.76E^{-8}$	8.4	$>1.00E^{-16}$
3	1.2	0.243	0.8	0.411	4.6	$3.72E^{-6}$
4	0.1	0.939	9.5	$>1.00E^{-16}$	13.8	$>1.00E^{-16}$
5	6.0	$1.79E^{-9}$	7.8	$8.77E^{-15}$	11.9	$>1.00E^{-16}$
6	1.0	0.327	15.7	$>1.00E^{-16}$	10.0	$>1.00E^{-16}$
7	3.7	$2.30E^{-4}$	12.1	$>1.00E^{-16}$	12.1	$>1.00E^{-16}$
8	3.1	0.002	8.5	$>1.00E^{-16}$	12.0	$>1.00E^{-16}$
9	1.3	0.204	9.5	$>1.00E^{-16}$	10.5	$>1.00E^{-16}$

Table S3. Statistical significances of biological responses represented as Z-Scores and equivalent p-values for FCRT Calu6 tumor and model.

tumor	V1-V2 Z	V1-V2 P	V1-V3 Z	V1-V3 P	V1-V2-V3 Z	V1-V2-V3 P
FCRT						
1	3.1	0.002	7.9	$>1.00E^{-16}$	8.5	$>1.00E^{-16}$
2	0.6	0.520	4.5	$5.49E^{-6}$	4.2	$2.58E^{-5}$
3	3.9	$9.11E^{-5}$	11.6	$>1.00E^{-16}$	9.8	$>1.00E^{-16}$
4	3.0	0.003	10.7	$>1.00E^{-16}$	8.8	$>1.00E^{-16}$
5	3.0	0.003	11.0	$>1.00E^{-16}$	12.3	$>1.00E^{-16}$
6	6.5	$1.04E^{-10}$	11.9	$>1.00E^{-16}$	5.7	$1.56E^{-8}$

Table S4. Statistical significances of biological responses represented as Z-Scores and equivalent p-values for ATV Calu6 tumor and model.

tumor	V1-V2 Z	V1-V2 P	V1-V3 Z	V1-V3 P	V1-V2-V3 Z	V1-V2-V3 P
ATV						
1	1.4	0.166	1.1	0.287	0.0	0.974
2	0.3	0.777	6.1	$1.13E^{-9}$	3.8	$1.72E^{-4}$
3	2.0	0.047	2.3	0.021	1.1	0.257
4	12.0	$>1.00E^{-16}$	7.1	$1.23E^{-12}$	10.9	$>1.00E^{-16}$
5	2.7	0.007	7.3	$2.56E^{-13}$	3.1	0.002
6	4.8	$1.53E^{-6}$	4.0	$6.88E^{-5}$	6.5	$7.41E^{-11}$
7	3.1	0.002	2.9	0.004	4.0	$7.70E^{-5}$
8	4.7	$2.39E^{-6}$	3.6	$3.36E^{-4}$	5.4	$6.03E^{-8}$
9	5.2	$1.75E^{-7}$	6.2	$6.93E^{-10}$	6.5	$7.22E^{-11}$
10	0.0	1.000	0.9	0.393	1.2	0.230
11	2.3	0.022	3.1	0.002	4.8	$1.80E^{-6}$
12	11.8	$>1.00E^{-16}$	10.6	$>1.00E^{-16}$	8.5	$>1.00E^{-16}$
13	1.5	0.132	1.9	0.053	1.3	0.194
14	0.1	0.912	4.3	$1.39E^{-5}$	3.2	0.001

Table S5. Statistical significances of biological responses represented as Z-Scores and equivalent p-values for additional CT26 tumors and models.

		Control			RT	
	Eff (%)	Z	P	Eff (%)	Z	P
CT26						
1	3.90±4	1.04	0.296	40.07±14	2.84	0.004
2	2.15±8	0.28	0.777	24.73±13	1.88	0.059
3	3.24±6	0.50	0.617	0.00±4	0.00	0.999
4	6.27±4	1.48	0.138	67.29±14	4.69	$2.73E^{-6}$
5	4.23±7	0.59	0.553	57.92±16	3.68	$2.30E^{-4}$
6	1.51±9	0.17	0.868	37.79±14	2.72	0.006
7	6.61±17	0.39	0.697	20.97±8	2.56	0.010
8	6.34±7	0.92	0.360	24.37±20	1.24	0.213
9	6.35±8	0.79	0.428	51.92±12	4.38	$1.16E^{-5}$
10	10.60±5	2.07	0.038	52.32±16	3.25	$1.17E^{-3}$
11	21.86±13	1.64	0.102	46.69±15	3.04	$2.39E^{-3}$
12	10.85±13	0.86	0.391	61.61±9	6.50	$8.05E^{-11}$
13	2.77±11	0.24	0.809	61.31±11	5.49	$3.98E^{-8}$
14	1.80±2	0.83	0.409	15.96±17	0.96	0.337
15	9.97±15	0.67	0.503	31.89±18	1.77	0.076
16	35.43±16	2.18	0.030	40.22±8	5.21	$1.89E^{-7}$
17	7.12±7	0.97	0.333	32.25±13	2.49	0.012
18	24.14±12	2.09	0.036	24.65±19	1.30	0.195
19	4.46±14	0.31	0.756	18.63±5	3.65	$2.66E^{-4}$
20	12.10±14	0.89	0.374	33.72±6	5.43	$5.72E^{-8}$
21	8.49±6	1.38	0.168	38.24±10	3.85	$1.19E^{-4}$
22	18.13±12	1.52	0.129	14.98±13	1.19	0.232

Table S6. Statistical significances of biological responses represented as Z-Scores and equivalent p-values for additional 4T1 tumors and models.

	Control			RT		
	Eff (%)	Z	P	Eff (%)	Z	P
4T1						
1	0.00±0	0.00	1.00	57.35±20	2.91	0.003
2	0.00±0	0.00	1.00	41.94±7	5.64	$1.66E^{-8}$
3	0.00±2	0.00	1.00	65.01±18	3.70	$2.12E^{-4}$
4	0.01±1	0.02	0.987	0.00±3	0.00	1.000
5	1.56±7	0.22	0.828	4.82±11	0.46	0.647
6	11.28±8	1.29	0.198	0.01±10	0.00	0.999
7	1.43±14	0.10	0.917	40.44±16	2.58	0.009
8	0.02±10	0.00	0.998	51.79±13	3.93	$8.38E^{-5}$
9	0.03±2	0.01	0.989	17.95±14	1.26	0.205
10	1.72±9	0.19	0.852	25.86±9	2.85	0.004

Table S7. Statistical significances of biological responses represented as Z-Scores and equivalent p-values for additional U87 tumors and models.

	Control			RT		
	Eff (%)	Z	P	Eff (%)	Z	P
U87						
1	3.16±4	0.72	0.474	40.15±6	6.90	$5.12E^{-12}$
2	2.47±6	0.41	0.682	49.70±10	5.09	$3.59E^{-7}$
3	6.06±8	0.80	0.425	48.60±7	7.35	$1.95E^{-13}$
4	2.87±7	0.43	0.668	54.57±6	9.24	$>1.00E^{-16}$
5	0.03±6	0.01	0.996	43.98±8	5.35	$8.86E^{-8}$
6	10.02±8	1.33	0.185	38.13±12	3.29	$9.94E^{-4}$
7	1.25±12	0.10	0.920	62.55±7	9.40	$>1.00E^{-16}$
8	0.00±0	0.0	1.000	79.69±8	10.55	$>1.00E^{-16}$
9	7.23±6	1.20	0.232	66.02±8	8.16	$>1.00E^{-16}$
10	0.91±6	0.16	0.874	7.76±8	0.96	0.337
11	17.15±6	2.91	0.004	32.11±13	2.49	0.012
12	4.97±7	0.73	0.468	23.30±11	2.03	0.042

Table S8. Statistical significances of biological responses represented as Z-Scores and equivalent p-values for additional HCT116 tumors and models.

	Control			RT		
	Eff (%)	Z	P	Eff (%)	Z	P
HCT116						
1	12.06±5	2.28	0.023	97.58±6	15.35	$>1.00E^{-16}$
2	0.00±6	0.00	1.000	67.19±6	12.15	$>1.00E^{-16}$
3	3.10±7	0.41	0.681	78.89±7	11.02	$>1.00E^{-16}$
4	22.13±9	2.52	0.012	16.13±11	1.49	0.135
5	0.00±0	0.00	1.000	36.30±5	7.13	$9.78E^{-13}$
6	1.79±8	0.23	0.815	61.02±5	12.80	$>1.00E^{-16}$
7	0.30±9	0.03	0.974	26.01±6	4.62	$3.79E^{-6}$
8	16.23±12	1.31	0.189	64.04±7	9.40	$>1.00E^{-16}$
9	4.94±7	0.72	0.472	43.06±14	3.15	0.001
10	0.00±0	0.02	0.985	73.67±6	12.43	$>1.00E^{-16}$
11	4.47±4	1.00	0.316	77.06±8	9.70	$>1.00E^{-16}$
12	9.84±10	1.00	0.320	49.96±7	7.00	$2.48E^{-12}$
13	4.62±3	1.39	0.165	53.37±11	4.80	$1.61E^{-6}$
14				84.75±5	17.14	$>1.00E^{-16}$
15				84.15±7	11.24	$>1.00E^{-16}$
